# Inferred calcification rate of a Mediterranean azooxanthellate coral is uncoupled with sea surface temperature along an 8° latitudinal gradient

**DOI:** 10.1186/1742-9994-9-32

**Published:** 2012-11-19

**Authors:** Erik Caroselli, Guido Mattioli, Oren Levy, Giuseppe Falini, Zvy Dubinsky, Stefano Goffredo

**Affiliations:** 1 Marine Science Group, Department of Biological, Geological and Environmental Sciences, Alma Mater Studiorum, University of Bologna, Via F. Selmi 3, Bologna, EU, 40126, Italy; 2 Operative Unit of Radiology and Diagnostics by Images, Hospital of Porretta Terme, Local Health Enterprise of Bologna, Via Roma 16 Porretta Terme, Bologna, EU, 40046, Italy; 3 The Mina and Everard Goodman Faculty of Life Sciences, Bar-Ilan University, Ramat-Gan, 52900, Israel; 4 Department of Chemistry “G. Ciamician”, Alma Mater Studiorum, University of Bologna, Via F. Selmi 2, Bologna, EU, 40126, Italy

**Keywords:** Asymbiotic coral, Coral growth, Dendrophylliidae, Global warming, Scleractinia, Temperate coral

## Abstract

**Introduction:**

Correlations between sea surface temperature (SST) and growth parameters of the solitary azooxanthellate Dendrophylliid *Leptopsammia pruvoti* were assessed along an 8° latitudinal gradient on western Italian coasts (Mediterranean Sea), to check for possible negative effects of increasing temperature as the ones reported for a closely related, sympatric but zooxanthellate species.

**Results:**

Calcification rate was correlated with skeletal density but not with linear extension rate, indicating that calcium carbonate deposition was preferentially allocated to keep a constant skeletal density. Unlike most studies on both temperate and tropical zooxanthellate corals, where calcification rate is strongly related to environmental parameters such as SST, in the present study calcification rate was not correlated with SST.

**Conclusions:**

The lower sensitivity of *L. pruvoti* to SST with respect to other sympatric zooxanthellate corals, such as *Balanophyllia europaea*, may rely on the absence of a temperature induced inhibition of photosynthesis, and thus the absence of an inhibition of the calcification process. This study is the first field investigation of the relationship between SST and the three growth parameters of an azooxanthellate coral. Increasing research effort on determining the effects of temperature on biological traits of the poorly studied azooxanthellate scleractinians may help to predict the possible species assemblage shifts that are likely to occur in the immediate future as a consequence of global climatic change.

## Introduction

Latitude is the main factor influencing the variation of light and temperature [1], two environmental parameters strongly linked to coral growth, physiology, demography and distribution pattern [2,3]. As a general trend, coral growth decreases with increasing latitude until a limit is reached where coral reef development no longer occurs, beyond 30°N and 30°S [4]. Coral growth can be defined by three related parameters (calcification = linear extension x skeletal density; [3,5]) whose measurement is essential when assessing the environmental effects on coral growth, because none of the three can perfectly predict the other two [6]. Analyzing these variables also allows predicting the possible effect of climatic changes on coral ecosystems [7,8]. These three variables have been studied in the field in the tropical genera *Montastraea*[5], *Diploastrea*[9], and *Porites*[3,7,8,10], and their variation has been linked to changes in sea surface temperature (SST) and light associated with time and latitude. In colonies of *M. annularis* of the Gulf of Mexico and the Caribbean Sea, SST is positively correlated with calcification rate and skeletal density, while it is negatively correlated with linear extension rate [5]. In colonies of *Porites* of the Hawaiian archipelago, Thailand, and the Great Barrier Reef (Australia) SST is positively correlated with calcification and linear extension rates, and negatively correlated with skeletal density [5]. In contrast, monitoring efforts of 16 years of calcification in *Porites* colonies from the Great Barrier Reef [7] and 21 years of calcification in *Porites* colonies from Thailand [8] show that calcification declined over time, and suggests that the response may be due to the interactive effects of elevated seawater temperatures and *p*CO_2_ increase, as previously reported for colonies of *Stylophora pistillata* grown in aquaria [11]. However, a recent analysis of calcification of *Porites* colonies along an 11° latitudinal gradient along Western Australia coasts has found no widespread patterns of decreasing calcification since 1900, and concludes that the main driver of change in coral calcification is the rate of change in the thermal environment of coral reefs [10].

In contrast with the large number of studies about the relationships between environmental parameters and coral growth in the tropics [3,5,7,8,10], such studies are scarce for temperate zones. In *Astrangia danae* and *Plesiastrea versipora*, calcification rate increases with temperature, similarly to some tropical corals, albeit over a lower temperature range [12]. Laboratory observations on calcification rates in *Cladocora caespitosa* and *Oculina patagonica* show that long periods of elevated temperatures, corresponding to or higher than the maximum summer temperature in the field, lead to a decrease of calcification [13].

This study investigated the relationships between SST and the three growth components (calcification, skeletal density, and linear extension) in the temperate/subtropical coral *Leptopsammia pruvoti* Lacaze-Duthiers, 1897. *Leptopsammia pruvoti* is an ahermatypic, non-zooxanthellate and solitary scleractinian coral, widely distributed in the Mediterranean basin and along the European Atlantic coast from Portugal through Southern England and Ireland [14]. It is one of the most common organisms in semi-enclosed rocky habitats, under overhangs, in caverns and small crevices at 0–70 m depth [14]. SST and solar radiation along an 850-km latitudinal gradient on Western Italian coasts do not significantly influence its population abundance or skeletal architecture features such as corallite length, width, height [15], or its population structure stability and demographic traits [16]. However, the density of calcium carbonate crystals composing its skeleton (micro-density; [17]) is positively correlated with SST [18]. It is a gonochoric internal brooder [19], with a genetic structure characterized by heterozygote deficits at all scales, from patch to populations, without correlations between genetic differentiation and geographic distance, and with most genetic differentiation occurring between patches of the same study site, rather than between sites [20]. Its bright yellow colour and abundance makes this species attractive to recreational divers, who represent an important income for coastal tourist resorts in the Mediterranean Sea [21].

This is the first study on the variation of the three growth components in an azooxanthellate coral, and it aims to assess the variations of calcification rate, linear extension rate, and skeletal density in populations arranged along a latitudinal SST gradient. The results are also considered in the light of the most recent climate change scenarios and compared to the findings on the zooxanthellate Mediterranean endemic Dendrophylliiid coral *Balanophyllia europaea*.

## Results

Mean annual SST varied significantly among the sites (Kruskal-Wallis test, *p* < 0.001; Table 1). Mean skeletal density, linear extension and calcification rates were significantly different among the populations of *Leptopsammia pruvoti* (Kruskal-Wallis test, *p* < 0.001; Table 2). To facilitate comparisons with published studies, the dependent and independent variables for the linear regression analyses between growth parameters (Table 3) were chosen according to literature data [3,5,22]. Mean skeletal density and calcification rate of the corallites in the populations were not correlated with mean linear extension rate (Table 3). Mean calcification rate of the corallites in the populations was positively correlated with mean skeletal density (Table 3). Based on the bootstrapping coefficients, calcification rate explained 67% of the variance in skeletal density (Table 3). 

**Table 1:** Average annual solar radiation and SST values of the sample sites

**Population**	**Code**	**SST (°C) annual mean (SE)**
Calafuria	CL	18.02 (0.04)
Elba	LB	18.74 (0.04)
Palinuro	PL	19.14 (0.03)
Scilla	SC	19.54 (0.02)
Genova	GN	19.56 (0.04)
Pantelleria	PN	19.88 (0.04)

The sites are arranged in order of increasing SST.

**Table 2:** ***Leptopsammia pruvoti*. Mean skeletal density, linear extension, and calcification rates values of the populations**

**Population**	**Code**	***n***	**Average skeletal density (mg mm**^**-3**^**)**	**SE**	**Average linear extension rate (mm yr**^**-1**^**)**	**SE**	**Average calcification rate (mg mm**^**-2**^**yr**^**-1**^**)**	**SE**
Calafuria	CL	210	1.56	0.07	0.79	0.01	1.26	0.06
Elba	LB	76	1.07	0.07	0.61	0.02	0.76	0.06
Palinuro	PL	152	1.38	0.08	0.74	0.01	1.14	0.06
Scilla	SC	115	1.50	0.07	0.69	0.02	1.00	0.05
Genova	GN	123	1.31	0.09	0.61	0.01	1.08	0.07
Pantelleria	PN	144	1.14	0.03	0.68	0.01	0.71	0.01

The sites are arranged in order of increasing SST. *n* number of individuals, SE standard error.

**Table 3:** *Leptopsammia pruvoti*

**Dependent variable**	**Independent variable**	**Slope (SE)**	**Intercept (SE)**	***r***^***2***^	***r***	***r***^***2***^_***BS***_	***r***_***BS***_
Skeletal density	Linear extension	-	-	0.518	0.720	0.457	0.676
Calcification	Linear extension	-	-	0.396	0.629	0.347	0.589
Calcification	Skeletal density	0.969	−0.294	0.753	0.868*	0.666	0.816*

Linear regression and correlation analysis between mean skeletal density, linear extension rate, and calcification rate in the six sites (*n* = 6). *r*^*2*^ Pearson’s coefficient of determination, *r* Pearson’s correlation coefficient, *r*^*2*^_*BS*_ and *r*_*BS*_ Pearson’s coefficients calculated with bootstrapping, * *p* < 0.05. SE standard error.

Considering the whole dataset (all ages), both the linear and power function models showed that none of the mean growth parameters of the populations were correlated with SST (Tables 4, 5). The lack of trends from the whole dataset was confirmed by the age-stratified analyses on the subsets of immature, mature, and old samples (Tables 4, 5). Thus, the mean growth parameters significantly differed among study sites, but their variation was not related to SST.

**Table 4:** *Leptopsammia pruvoti*

**Dependent variable**	***r***^***2***^	***r***	***r***^***2***^_***BS***_	***r***_***BS***_
All samples				
Skeletal density	0.126	−0.355	0.073	−0.271
Linear extension	0.250	−0.500	0.143	−0.378
Calcification	0.261	−0.510	0.181	−0.426
Immature samples (0–4 years)				
Skeletal density	0.018	−0.133	0.0003	−0.018
Linear extension	0.013	−0.115	0.017	−0.129
Calcification	0.178	−0.422	0.129	−0.359
Mature samples (5–8 years)				
Skeletal density	0.049	−0.221	0.030	−0.174
Linear extension	0.077	−0.278	0.014	−0.118
Calcification	0.016	−0.126	0.008	−0.090
Old samples (>8 years)				
Skeletal density	0.301	0.548	0.274	0.523
Linear extension	0.148	−0.384	0.122	−0.349
Calcification	0.181	0.425	0.091	0.302

Linear model. Correlation analysis between SST and growth parameters in the six sites (*n* = 6). No correlation resulted significant. *r*^*2*^ Pearson’s coefficient of determination, *r* Pearson’s correlation coefficient, *r*^*2*^_*BS*_ and *r*_*BS*_ Pearson’s coefficients calculated with bootstrapping.

**Table 5:** *Leptopsammia pruvoti*

**Dependent variable**	***r***^***2***^	***r***	***r***^***2***^_***BS***_	***r***_***BS***_
All samples				
Skeletal density	0.105	−0.324	0.067	−0.258
Linear extension	0.225	−0.474	0.133	−0.365
Calcification	0.223	−0.472	0.165	−0.406
Immature samples (0–4 years)				
Skeletal density	0.032	−0.178	0.0004	−0.022
Linear extension	0.013	−0.114	0.012	−0.111
Calcification	0.204	−0.451	0.123	−0.351
Mature samples (5–8 years)				
Skeletal density	0.051	−0.225	0.021	−0.146
Linear extension	0.077	−0.277	0.013	−0.112
Calcification	0.014	−0.119	0.004	−0.065
Old samples (>8 years)				
Skeletal density	0.299	0.546	0.299	0.546
Linear extension	0.143	−0.378	0.113	−0.336
Calcification	0.268	0.518	0.144	−0.380

Power function model (eqn 2). Linear regression and correlation analysis between SST and growth parameters in the six sites (*n* = 6) calculated on log-transformed data. No correlation resulted significant. *r*^*2*^ Pearson’s coefficient of determination, *r* Pearson’s correlation coefficient, *r*^*2*^_*BS*_ and *r*_*BS*_ Pearson’s coefficients calculated with bootstrapping.

## Discussion

The ‘stretching modulation of skeletal growth’ is a mechanism allowing corals to preferentially invest calcification resources in thickening the skeleton, thus increasing skeletal density, or accelerating linear extension [5,23]. The tropical *Porites*, for example, invests increased calcification at higher temperatures into linear extension [3,8]. In contrast, the tropical *Montastraea annularis* invests increased calcification at higher temperatures to construct denser skeletons [5,23]. In the Mediterranean endemic *Balanophyllia europaea*, calcification is allocated evenly between increasing skeletal density and linear extension, indicating that the ability to colonize the substratum quickly and the mechanical strength of the skeleton are both important for this species [22]. The temperate *L. pruvoti* exhibited a response which was similar to the one of *M. annularis*, in that calcification was positively correlated with skeletal density but not with linear extension. For each 1 mg mm^-2^ yr^-1^ of calcification rate variation, skeletal density varied by ~ 1 mg mm^-3^.

Geometrically calculated skeletal density values in the present work were reasonable with respect to other studies on tropical and temperate species [3,5,17,22]. The computed skeletal density used in this and in previous studies [15,22] is analogous to the bulk density [17], which is defined as the skeletal mass divided by the total volume (skeletal matrix volume plus pores volume; [17]). Skeletal matrix volume is further composed by the crystals of CaCO_3_ and by the intracrystalline organic matrix regulating the crystallization process [24]. Analyses to quantify the porosity in the same samples of the present study show that the variation of bulk density depends on variations of porosity, while the variation in the density of the skeletal framework (micro-density, [17]) is not strong enough to significantly affect bulk density [18].

The lack of correlations with SST exhibited by the calcification rate and skeletal density in the present study on *Leptopsammia pruvoti* confirms previous studies on the population density, growth and population structure stability of this species, where the coral parameters were always shown to be unrelated to environmental variables such as solar radiation or SST [15,16]. For both the linear and power function models, trends of the analyses performed on the full dataset were confirmed by the analyses on the three age-based subsets, indicating that differences in the mean age of the samples in the populations [16] did not bias the results.

The lack of correlation between calcification rate of the azooxanthellate *L. pruvoti* and SST along the latitudinal gradient is a different response with respect to the similar studies on temperate and tropical zooxanthellate species. For example, calcification rate of the Mediterranean endemic *B. europaea* is negatively related to SST [22], while in the tropical *Porites* and *M. annularis* it is positively related to SST [3,5]. However, mid-term studies on *Porites* highlight a reduction of its calcification rate as SST increases [7,8], even if a recent long-term analysis of *Porites* calcification along Australian coasts show no evidence of widespread patterns of decline in calcification rate since 1900 [10]. In that analysis, calcification rates at high-latitude reefs were found to be more sensitive to temperature increase than more tropical reefs [10]. Another recent analysis of *Porites* spp. and *Montastraea* spp. in the Great Barrier Reef and Mexican Caribbean highlighted a negative response of calcification to increasing SST for both genera, but a higher sensitivity to temperature increase for the former genus, rather than the latter one [25]. This has fundamental consequences in light of future global warming scenarios, since differential reduction of calcification between coral genera could profoundly affect community structure [25]. Our results suggest a higher sensitivity of zooxanthellate species to the variations of temperature, while asymbiotic corals may be more tolerant to temperature variations. The higher sensitivity of symbiotic species may be due to the decrease of photosynthetic performance at higher temperatures, since in zooxanthellate corals calcification is enhanced by photosynthesis [26], and both processes have temperature optima [12]. Alternatively, a role may be played by the much steeper response of respiration to subtle temperature increases (Q_10_) than that of photosynthesis, resulting in significant decrease of the residual net photosynthesis and of the energy surplus needed for calcification and other physiological processes [27]. Although the hypothesis of photosynthetic inhibition at high temperatures is intriguing, other environmental parameters may influence coral calcification (pH, total alkalinity, wave exposition, flow rate, etc.). Besides local factors, the apparent insensitivity of *L. pruvoti* growth to the SST range experienced in the present study may be due either to 1) the lack of zooxanthellate, and thus a lack of inhibition of calcification by the depressed net photosynthesis, or 2) a higher optimal temperature for the calcification of this species with respect to *B. europaea*, or 3) a coupling between the above two factors, or 4) a sampling area not representative of the species conditions at the collection sites. *L. pruvoti* distribution area includes also regions outside the Mediterranean Sea, up to the southern coasts of Ireland and UK, where seawater temperature is considerably lower [14]. It is then unlikely that this species has a higher optimal temperature for calcification than the Mediterranean endemic *B. europaea*, since *L. pruvoti* lives in much colder seas and deeper waters (up to 70 m depth). Even if any comparison between *L. pruvoti* and *B. europaea* must be taken cautiously, since the two species were sampled at different depths (16 m and 6 m, respectively), which may be subject to different thermal regimes throughout the year, the variation of calcification rate among sites, found in *L. pruvoti*, could be related to particular local conditions unrelated to temperature. Since the present study focused on the influence of SST, we selected sites with similar environmental traits other than SST, but we did not thoroughly analyze all the site characteristics such as nutrients and zooplankton availability or competitive interactions with other organisms, which could all contribute to the observed differences in calcification rate. However, these local differences, while contributing to the variability of calcification rate (this study) and of population dynamics traits [16], are not strong enough to determine significant variations in population abundance, which is homogeneous across all sites with about 10,000 individuals per square meter [15]. It may be argued that no correlation with SST has been found because the selected sampling area for this study was too small and unrepresentative of the population. However, the same sampling area adequately represents the sites in previous studies on the biometry, growth and population dynamics of the species [15,16,22], where trends in the biometric parameters (such as polyp length) with temperature have been found [15]. Moreover, significant differences in calcification rate among sites have actually been found in the present study, but they do not correlate to temperature, and are likely due to local differences in parameters other than temperature. An alternative explanation of the difference in demographic parameters among sites may be related to suspension feeding. In the Mediterranean, the warm summer–fall season is characterized by lower nutrient levels and zooplankton availability than the cool winter–spring season [28]. Corals and several benthic suspension feeding taxa have proved to be stressed by low nutrients and limited zooplankton availability [28]. Different availability of resources among sites may affect calcification rate in *L. pruvoti*. However, if this was the case, negative effects on calcification rate would be expected in the warmest sites (where the warm season is longer and the zooplankton availability lower, on average). Instead, *L. pruvoti* calcification seems to be unrelated to SST. The differences in calcification rates and population dynamics traits among sites may be related to other environmental parameters not considered in this study (pH, total alkalinity, wave exposition, flow rate, etc.). Further investigations are thus needed to better constrain the environmental controls on the population dynamics of this species. Moreover, further investigation on the poorly studied azooxanthellate species are needed to differentiate the environmental controls on the growth of symbiotic and asymbiotic corals.

One of the main threats for coral and coral reefs survival is global temperature increase [29]. The speeds of many negative changes to the oceans are near or are tracking the worst-case scenarios from the IPCC and other predictions [30]. Recently, one of the most diverse communities in the Mediterranean Sea, the coralligenous (~1,666 species; [31]), where suspension feeders are dominant, has been strongly affected by several mass mortality events related to high temperatures [32-36]. The zooxanthellate dendrophylliid *B. europaea* is a Mediterranean endemic species which will likely be negatively affected by seawater warming, since increasing temperature lowers its population abundance, its skeletal density [15], by increasing its skeletal porosity [18], and lowers its calcification rate [22]. Moreover, warmer populations are less stable and show a progressive deficiency of young individuals, so that there is concern for the future of this species [37]. These detrimental effects of increasing temperature seem to be related to the symbiosis with zooxanthellae, whose photosynthesis could be depressed at high temperatures causing cascading negative effects on the growth and reproductive traits of *B. europaea*, although this hypothesis is yet to be tested [15,18,22,37]. *L. pruvoti*, instead, seems to be tolerant to the same temperature range experienced by *B. europaea.* In fact, biological traits of the former species have been studied in the same sites and time interval, but none of them is negatively correlated with SST ( [15,16,18] and present study). Increasing temperature may even favour *L. pruvoti*, since the corals living in populations characterized by higher SSTs have a higher micro-density, even if this increase in micro-density is not strong enough to cause significant variations of bulk density [18]. However, the limit of temperature increase that will still be tolerable by this species is unknown. Moreover, it should be noted that the results derived from analyses based on latitudinal variations of calcification are not necessarily the same as those derived from time-based analyses. In fact, while calcification may have a positive correlation with SST along a latitudinal gradient, such as in *Porites*[3], it may be negatively correlated with the increasing SST recorded in recent years [7,8], and may fluctuate during the yearly cycle of temperature variation [38]. Thus, any extrapolations of spatial derived data to time resolved predictions has to be taken cautiously.

## Conclusions

Unlike the zooxanthellate *B. europaea*, the differences in growth and population dynamics traits of the azooxanthellate *L. pruvoti* seem unrelated to SST along a wide latitudinal gradient in the Mediterranean Sea. These findings confirm previous observations that two species belonging to the same family and sharing a wide part of their distribution area may have very different temperature tolerance and consequent response to seawater warming [16]. The higher tolerance of *L. pruvoti*, relative to *B. europaea*, may indeed rely on the absence of symbionts, and thus the lack of an inhibition of host physiological processes by the heat-stressed zooxanthellae.

This study is the first field investigation of the relationship between SST and the three growth parameters of an azooxanthellate coral. Increasing research effort on determining the effects of temperature on biological traits of the poorly studied azooxanthellate scleractinians may help to predict the possible species assemblage shifts that are likely to occur in the immediate future as a consequence of global climatic change.

## Materials and methods

**Figure 1:**
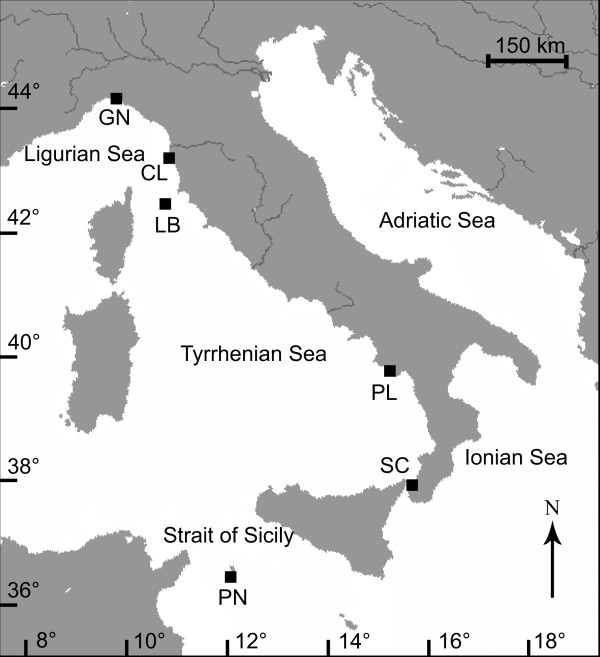
**Map of the Italian coastline indicating sites where corals were collected.** Abbreviations and coordinates of the sites in decreasing order of latitude: GN Genova, 44°20'N, 9°08'E; CL Calafuria, 43°27'N, 10°21'E; LB Elba Isle, 42°45'N, 10°24'E; PL Palinuro, 40°02'N, 15°16'E; SC Scilla, 38°01'N, 15°38'E; PN Pantelleria Isle, 36°45'N, 11°57'E.

Specimens of *Leptopsammia pruvoti* were collected from six sites along a latitudinal gradient, from 44°20'N to 36°45'N, between 9 November 2003 and 30 September 2005 (Figure 1). Sampling sites were selected along the gradient to be characterized by different SST, which is the environmental parameter considered in this study and that has already shown correlations with biologic parameters of *L. pruvoti* in previous studies [15,18]. Samples were collected in each site using transects of three triangular patches of base × height equal to 12 cm × 7.1 cm (single patch area = 42.6 cm^2^; transect area per each site = at least 42.6 × 3 = at least 128 cm^2^; [16]). Triangular patches were more easily placed in the narrow crevices colonized by the species, with respect to traditional square patches. Such a small patch area was chosen because of the high population density of the species (about 10,000 individuals m^-1^) which makes the sampling of all individuals present in larger areas (such as 1 m^2^) unfeasible [15]. Moreover, such sampling area is considered representative of the studied site in previous studies of the biometry, growth and population dynamics of this species, where significant differences among sites and correlations with SST have been found [15,16,39]. Sampling was performed at depths known to have high population densities and where the reproductive biology, biometry, population density, growth, population dynamics, and genetics of the species had previously been studied [15,16,19,20,39]. Patches were collected on the vault of crevices 3 m apart, at a depth of 15–17 m. All crevices were clearly separated one from each other, without a continuous presence of polyps from one patch to each other. In order to account for the high within-site genetic variation characterizing the species [20], it was necessary to sample different patches at each site and treat them as replicates, to have a meaningful picture of the growth parameters at each site. Because of the random distribution pattern of the species, the problems associated with regularly spaced quadrats and transects do not apply to this study [15]. All of the polyps present in each patch were collected. 

Corals were dried at 50°C for four days and observed under a binocular microscope to remove fragments of substratum and calcareous deposits produced by other organisms. Corallite length (*L*: maximum axis of the oral disc), width (*W*: minimum axis of the oral disc) and height (*h*: oral-aboral axis) were measured with calipers and the dry skeletal mass (*M*) was measured with a precision balance. Corallite volume (*V*) was determined by applying the formula: *V*=*L*^2^x*W*^2^x*h*π [15]. Skeletal density (*D*) was calculated by dividing *M* by *V*.

The age of each sample was estimated using the von Bertalanffy length-age growth function previously obtained and based on growth bands analysis by means of computerized tomography [37,40]. According to the age of the polyp, the annual linear extension rate was obtained for each sample using the von Bertalanffy length-age growth function [16,40]. The mean annual calcification rate (mass of CaCO_3_ deposited per year per area unit) was calculated for each sample by the formula: calcification (mg mm^-2^ yr^-1^) *=* skeletal density (mg mm^-3^) *x* linear extension (mm yr^-1^) [3,5,22]. Thus, for each population the mean values of skeletal density, linear extension and calcification rates of the corallites were obtained. Samples were divided into three age classes: immature (0–4 years, after [16]); mature (4–8 years, double the age at sexual maturity); old (>8 years).

Correlation and regression analyses between environmental and growth parameters were performed both for the full dataset and for the three age classes, to check for differences due to the different mean age of the samples in the populations [12]. Relationships between environmental and growth parameters were performed using two models: a linear model and a power function model. The linear model was used to compare the results with other studies on environmental controls of coral growth, where linear functions are used [3,5]. Also the power function model was used as it produced the best fit with the data, and to compare the results obtained by the linear model. The power function model:

(1) $$y=\text{a}x^{\text{b}}$$

was linearized with a log-transformation of both the independent and dependent variables, producing the equation:

(2) $$\text{ln}\left(y\right)=\text{bln}\left(x\right)+\text{ln}\left(\text{a}\right)$$

SST data for 2003–2005 were obtained for each location from the National Mareographic Network of the Agency for the Protection of the Environment and Technical Services (APAT, now renamed to Superior Institute for Environmental Research Protection, ISPRA, [41]). The data are measured by mareographic stations SM3810, built by the Italian Society for Precision Apparatuses (SIAP). Mean annual SST was obtained from hourly values measured from January 2001 to January 2005 (Table 1).

Because of the heteroskedastic nature of the data, the non-parametric Kruskal-Wallis test was used to compare mean SST, skeletal density, linear extension and calcification rates among the populations. Pearson correlation coefficients were calculated for the relationships among growth parameters and between environmental and growth parameters. Because of the low *n* value (*n* = 6) and the assumptions of the Pearson method, correlation coefficients were also estimated with bootstrapping [42], with 100,000 resamples. All analyses were computed using PASW 18.0.

## Competing interests

The authors declare that they have no competing interests.

## Authors’ contributions

SG conceived and designed the experiments. EC and GM performed the experiments. EC analyzed the data. EC, GM, OL, GF, ZD and SG wrote the paper. All authors read and approved the final manuscript.
